# Preface to the special issue “Synthetic Biology and Bioproduction in Plants”

**DOI:** 10.5511/plantbiotechnology.24.0000p

**Published:** 2024-09-25

**Authors:** Nobutaka Mitsuda, Hikaru Seki, Tsubasa Shoji, Masami Yokota Hirai

**Affiliations:** 1 Bioproduction Research Institute, National Institute of Advanced Industrial Science and Technology (AIST), Sapporo, Hokkaido 062-8517, Japan; 2 Global Zero Emission Research Center, National Institute of Advanced Industrial Science and Technology (AIST), Tsukuba, Ibaraki 305-8566, Japan; 3 Department of Biotechnology, Graduate School of Engineering, Osaka University, Suita, Osaka 565-0971, Japan; 4 Industrial Biotechnology Initiative Division, Institute for Open and Transdisciplinary Research Initiatives, Osaka University, Suita, Osaka 565-0871, Japan; 5 Institute of Natural Medicine, University of Toyama, Toyama, Toyama 930-0194, Japan; 6 RIKEN Center for Sustainable Resource Science, Yokohama, Kanagawa 230-0045, Japan; 7 Department of Applied Biosciences, Graduate School of Bioagricultural Sciences, Nagoya University, Nagoya, Aichi 464-8601, Japan

Synthetic biology is a new biotechnological trend involving the transfer of genetic circuits, typically of multiple genes from certain organisms or generated artificially, leading to the creation of novel organisms. A typical example of synthetic biology is “Golden Rice”, which is engineered to produce high amounts of β-carotene in rice grains by introducing genes derived from other plants and bacteria (Paine et al. 2005; Peter et al. 2002). This special issue focuses on synthetic biology and bioproduction in plants. It includes six reviews, one mini review, four original papers, two short communications, and two notes related to this area of expertise.

Hayakawa et al. (2024) explains the definition, history, and market prospects of synthetic biology. This includes several examples of plant synthetic biology studies that are related to nutritional components, photosynthesis, lignocellulose, secondary metabolism, biosensors, nitrogen fixation, and auto-luminescent plants. Yoshida et al. (2024) outlines strategies for lignocellulose bioengineering in plants by redesigning the lignin and cell wall glycan pathways and explains the structural basis for lignocellulose recalcitrance. Shirai (2024) reviews metabolic design techniques adapted in synthetic biology and explores their potential applications in plant bioproduction. The review emphasizes the importance of optimally designing a comprehensive system of metabolic processes encompassing carbon flow, energy production and consumption, and redox balance for efficient bioproduction. These three articles clearly articulate the concept of synthetic biology and its applications in plants.

Revealing the biosynthetic pathways of plant metabolites is an important topic in the synthetic biology field. Phenylethanoid glycosides are a group of specialized metabolites mainly derived from plants of the Lamiales order. Fuji et al. (2024) reviewed glycoside distribution, biosynthesis, and bioproduction, particularly focusing on acteoside, which exists in over 150 species. *Lithospermum erythrorhizon* produces red naphthoquinone pigments: shikonin and alkannin. Manabe et al. (2024) conducted a feeding assay with labeled intermediates to clarify the shikonin and alkannin biosynthetic route in *L. erythrorhizon* cell cultures. *Corydalis* plants from the Papaveraceae family produce medicinal benzylisoquinoline alkaloids. Yamada et al. (2024a) discovered a set of highly expressed genes in tubers encoding metabolic enzymes, including two *O*-methyltransferases essential for alkaloid biosynthesis.

The bioproduction of useful compounds in plants requires an understanding of the regulatory mechanisms of their biosynthesis. Two clade Ia bHLH transcription factors are involved in the regulation of bioactive triterpenoid biosynthesis: BpbHLH9 from *Betula platyphylla* and LjbHLH50 from *Lotus japonicus*. *BpbHLH9* overexpression enhances the production of betulinic acid and oleanolic acid triterpenoids in *L. japonicus* hairy roots and upregulates *LjbHLH50* expression, suggesting a bHLH self-activation mechanism (Suzuki et al. 2024). Tamura et al. (2024) constructed a manually curated database of metabolic enzymes and transcription factors involved in plant triterpenoid biosynthesis that helps understand the regulatory mechanism of triterpenoid biosynthesis.

Synthetic biology typically involves manipulating plant metabolic pathways, followed by their introduction into easy-to-cultivate or model plants, such as *Nicotiana benthamiana*. Corosolic acid (often referred to as “phyto-insulin”) is a pentacyclic triterpenoid with insulin-like properties. Romsuk et al. (2024) engineered a metabolic pathway to produce this therapeutic triterpenoid by transiently expressing genes encoding several metabolic enzymes in *N. benthamiana*, including CYP716As and their electron-transfer partner CYP reductase. Hydrolyzable tannins are a group of polyphenols with defensive properties. The biosynthesis of polyphenols is mediated by undefined acyltransferases and oxidases and attempts have been made to reconstruct the pathway in heterogenous plants (Tahara et al. 2024). Steroidal glycoalkaloids are toxic specialized metabolites produced in potato that are linked to food poisoning. Low-toxicity potatoes were created using transcription activator-like effector nuclease (TALEN)-based genome editing by disrupting *CYP88B1*, a gene encoding a key enzyme in solanine biosynthesis in potato (Yasumoto et al. 2024).

The introduction of complete biosynthetic pathways elucidated from plants into microorganisms is a promising strategy. The integration of a co-culture system and transport engineering can be adapted to reconstruct a complex metabolic pathway in multiple cells (Yamada et al. 2024b). The glucose esters of phenolic acids (acyl-glucoses) are served as precursors for various acylated compounds or glucosides with potential health benefits. The expression of an ester-forming glycosyltransferase from sweet potato in *Escherichia coli* converts cinnamates and benzoates to their glucose esters (Kobayashi et al. 2024).

The bioproduction of pharmaceuticals in plants (molecular farming) is a big issue in the field. Fukuzawa et al. (2024) describes the advantages of plant-made pharmaceuticals, plant expression systems, host engineering, and cultivation techniques, especially focusing on therapeutic protein production, such as vaccines and antibodies. The coronavirus 2019 (COVID-19) pandemic has accelerated these studies in recent years.

Callus cultures have long been a fundamental experimental system in plant biotechnology, providing versatile platforms for plant propagation, genetic transformation, and metabolic engineering. Despite their widespread use, our understanding of the intrinsic properties and variability of plant callus cultures is limited. Multi-omics dataset analysis of metabolites, phytohormones, and transcripts from callus cultures of tobacco, rice, and bamboo highlights interspecies differences in metabolic potentials, indicating variable prospects of different callus cultures as bioproduction hosts (Kim et al. 2024).

It is projected that the global market for synthetic biology will increase to 35.7 billion USD by 2027 (Hayakawa et al. 2024), driven by improved genome editing and gene synthesis technologies, indicating significant opportunities for new challenges and investments. We believe that bioproduction in plants and the transfer of biosynthetic pathways from specific plants to other hosts using synthetic biology concepts will increasingly attract attention in terms of environmental protection, energy constraints, and the bioeconomy. We hope that this special issue will help to develop this field, together with plant biotechnology and other areas of science.

## Abbreviations

COVID-19: coronavirus disease 2019
TALEN: transcription activator-like effector nuclease
